# Supplementary material for: Presynaptic APP levels and synaptic homeostasis are regulated by Akt phosphorylation of huntingtin
Source: eLife. 2020 May 26;9:e56371. doi: 10.7554/eLife.56371 (PMC7269668; doi:10.7554/eLife.56371)
Supplement: Supplementary file 1. — Results are presented in percentages unless otherwise indicated. No significant differences between genotypes were observed. [file elife-56371-supp1.docx]

**Table 1:** The modified SHIRPA primary screen in HTT_SA_ mutant and controls (HTT_WT_)

| **Tests** | **WT (n=11)** | **HTT_SA_ (n=12)** |
| --- | --- | --- |
| **In the viewing Jar**  **(section1)** |  |  |
| Coat color | Normal: 11 (100%) | Normal: 12 (100%) |
| Hair length | Normal: 11 (100%) | Normal: 12 (100%) |
| Respiration rate | Normal: 11 (100%) | Normal: 12 (100%) |
| Tremor | None: 11 (100%) | None: 12 (100%) |
| Body position | Sitting or standing:  11 (100%) | Sitting or standing:  12 (100%) |
| Spontaneous activity | Vigorous scratch, groom,  moderate movement: 11 (100%) | Vigorous scratch, groom,  moderate movement: 12 (100%) |
| Defecation | None: 11 100%) | None: 10 (83%) |
|  | Done: 0 (0%) | Done: 2 (17%) |
| Urination | None: 11 (100%) | None: 10 (83%) |
|  | Done: 0 (0%) | Done: 2 (17%) |
|  |  |  |
| **In the Arena**  **(section 2)** |  |  |
| Elapsed time before the mouse starts to move (s) | Less than 3s: 11 (100%) | Less than 3s: 12 (100%) |
| Transfer arousal | Brief freeze (few sec), then  active movement: 11 (100%) | Brief freeze (few sec), then  active movement: 11 (100%) |
| Locomotor activity | Average number of squares  entered over 1 min exploration:  18.64 ± 2.402 | Average number of squares  entered over 1 min exploration:  21.50 ± 2.583 |
| Palpebral Closure | Eyes wide open: 11 (100%) | Eyes wide open: 12 (100%) |
| Piloerection | None: 11 (100%) | None: 12 (100%) |
| Gait | Normal: 11 (100%) | Normal: 12 (100%) |
| Startle Response | Preyer reflex (backwards  flick of pinnae): 11 (100%) | Preyer reflex (backwards  flick of pinnae): 12 (100%) |
| Pelvic Elevation | Normal (3mm elevation):  11 (100%) | Normal (3mm elevation):  12 (100%) |
| Tail Elevation | Horizontally extended:  11 (100%) | Horizontally extended:  12 (100%) |
| Touch Escape | Mild (escape response  to firm stroke):11 (100%) | Mild (escape response  to firm stroke): 12 (100%) |
| Tail morphology | Normal: 11 (100%) | Normal: 12 (100%) |
| Convulsions | Phenotype: 0 (0%) | Phenotype: 0 (0%) |

| **Tests** | **WT (n=11)** | **HTT_SA_ (n=12)** |
| --- | --- | --- |
| **On or Above the arena**  **(section 3)** |  |  |
| Wire manoeuver  (Horizontal bar) | Active grip  with hindlegs (5sec): 4 (36%) | Active grip  with hindlegs (5sec): 8 (67%) |
|  | Difficulty to grasp  with hindlegs: 2 (18%) | Difficulty to grasp  with hindlegs: 1 (8%) |
|  | Unable to grasp  with hindlegs: 3 (27%) | Unable to grasp  with hindlegs: 2 (17%) |
|  | Unable to lift hindlegs,  falls within seconds: 1 (9%) | Unable to lift hindlegs,  falls within seconds: 1 (8%) |
|  | Falls immediately: 1 (9%) | Falls immediately: 0 (0%) |
| Negative geotaxis  (vertical grids) | turns and climbs the grid:  11 (100%) | turns and climbs the grid:  12 (100%) |
| Positional Passivity :  struggles when held by tail | Yes: 11 (100%) | Yes: 12 (100%) |
| Trunk curl | Absence: 11 (100%) | Absence: 12 (100%) |
| Limb grasping | Present: 11 (100%) | Present: 12 (100%) |
| Visual Placing | Before vibrasse contact:  11 (100%) | Before vibrasse contact:  12 (100%) |
| Grip Strength | Moderate grip, effective:  11 (100%) | Moderate grip, effective:  12 (100%) |
| Body Tone | Slight resistance: 11 (100%) | Slight resistance: 12 (100%) |
| Head morphology | Normal: 11 (100%) | Normal: 12 (100%) |
| Corneal Reflex | Active single eye blink:  11 (100%) | Active single eye blink:  12 (100%) |
|  |  |  |
| **Supinate restraint**  **(section 4)** |  |  |
| Lacrimation | None: 11 (100%) | None: 12 (100%) |
| Whisker morphology | Normal: 11 (100%) | Normal: 12 (100%) |
| Tooth morphology | Normal: 11 (100%) | Normal: 12 (100%) |
| Provoked Biting | Present: 11 (100%) | Present: 12 (100%) |
| Salivation | None: 11 (100%) | None: 12 (100%) |
| Heart Rate | Normal: 11 (100%) | Normal: 12 (100%) |
| Abdominal tone | Slight resistance: 11 (100%) | Slight resistance: 12 (100%) |
| Skin color | Pink: 11 (100%) | Pink: 12 (100%) |
| Toe pinch | Slight withdrawal, not brisk:  5 (55%) | Slight withdrawal, not brisk:  0 (0%) |
|  | moderate withdrawal, not brisk:  6 (45%) | moderate withdrawal, not brisk:  12 (100%) |
