# Supplementary material for: Presynaptic APP levels and synaptic homeostasis are regulated by Akt phosphorylation of huntingtin
Source: eLife. 2020 May 26;9:e56371. doi: 10.7554/eLife.56371 (PMC7269668; doi:10.7554/eLife.56371)
Supplement: Transparent reporting form [file elife-56371-transrepform.docx]

***eLife’s* transparent reporting form**

We encourage authors to provide detailed information *within their submission* to facilitate the interpretation and replication of experiments. Authors can upload supporting documentation to indicate the use of appropriate reporting guidelines for health-related research (see [EQUATOR Network](http://www.equator-network.org/%20)), life science research (see the [BioSharing Information Resource](https://biosharing.org/)), or the [ARRIVE guidelines](http://www.plosbiology.org/article/info:doi/10.1371/journal.pbio.1000412) for reporting work involving animal research. Where applicable, authors should refer to any relevant reporting standards documents in this form.

If you have any questions, please consult our Journal Policies and/or contact us: [editorial@elifesciences.org](mailto:editorial@elifesciences.org).

**Sample-size estimation**

- You should state whether an appropriate sample size was computed when the study was being designed
- You should state the statistical method of sample size computation and any required assumptions
- If no explicit power analysis was used, you should describe how you decided what sample (replicate) size (number) to use

Please outline where this information can be found within the submission (e.g., sections or figure legends), or explain why this information doesn’t apply to your submission:

Figure 1 not applicable

Figure 2 B Sample size was estimated according to previous studies on axonal transport (Virlogeux et al,. 2018, Colin et al., 2008; Zala et al,. 2013)

Figures 2, 3 and 4 Sample size was estimated according to previous studies on axonal transport (Virlogeux et al,. 2018; Colin et al., 2008; Zala et al,. 2013) and preliminary result on Htt phosphorylation on APP axonal transport (minimum of 25 neurons and 100 vesicles analyzed to detect significant differences between WT and HTTSA groups). A minimum of 3 biological replicates were performed to control artifact.

Figure 5A For exocytosis rate of APP, sample size was empirically determined by significante differences determined using an unpaired t-test.

Figures 5B, 5C, 5D and 8B For westernblot analyses sample size was estimated according to statistical power a priori analysis using GPower software. For a power of 60%, a Wilcoxon Mann whitney test (two group) applied on preliminary results predicted a minimum of 5 replicates. This minimum was applied for all western blot analyses.

Figure 6 B: 4 littermate animals per genotype were analysed. This sample size was empirically determined by significant differences determined using a Mann-Whitney test.

Figures 6C and 8C : a minimum of 100 synapses analyzed by genotype was empirically determined according previous study (Zhang, Song et al., 2015) and experiments. A minimum of 3 biological replicates (mice) were performed to control artifact.

Figure 7 sample size was empirically determined by significante differences determined using an unpaired t-test or one way ANOVA. A minimum of 50 synatic contacts per condition was determined by this way.

Figure 9

Sample size were estimated according to previous studies (Martin et al. 2018 ; Lo et al. 2013 and Scholtzova et al. 2008) to meet ethical concerns in animal experimentation and scientific objectives. No explicit power analysis was used as most behavioral studies design in genetically modified organisms typically include 10 animals per group. Noticeably, working with aged mice is also a limiting factor for experimental designs.

**Replicates**

- You should report how often each experiment was performed
- You should include a definition of biological versus technical replication
- The data obtained should be provided and sufficient information should be provided to indicate the number of independent biological and/or technical replicates
- If you encountered any outliers, you should describe how these were handled
- Criteria for exclusion/inclusion of data should be clearly stated
- High-throughput sequence data should be uploaded before submission, with a private link for reviewers provided (these are available from both GEO and ArrayExpress)

Please outline where this information can be found within the submission (e.g., sections or figure legends), or explain why this information doesn’t apply to your submission:

We considered different populations of cells to be biological replicates.

All experiments were performed 3 times minimum for biological replication. This is 3 animals for brain samples (electronic microscopy, MRI, Westernblot…), 3 different cultures for cell culture, 3 different batches for behavior experiment.

Fourthy consecutive measurements on one mouse or on a culture of cells could be considered as 40 technical replicates.

Informations about replicates are provided in figure legends.

Behavioral measurements relying on cognitive tests are typically performed once for a specific task, hence information on replicates on the same animals do not apply to this type of data. Details about exclusion/inclusion criteria are provided in Materials and Methods when applicable.

**Statistical reporting**

- Statistical analysis methods should be described and justified
- Raw data should be presented in figures whenever informative to do so (typically when N per group is less than 10)
- For each experiment, you should identify the statistical tests used, exact values of N, definitions of center, methods of multiple test correction, and dispersion and precision measures (e.g., mean, median, SD, SEM, confidence intervals; and, for the major substantive results, a measure of effect size (e.g., Pearson's r, Cohen's d)
- Report exact p-values wherever possible alongside the summary statistics and 95% confidence intervals. These should be reported for all key questions and not only when the p-value is less than 0.05.

Please outline where this information can be found within the submission (e.g., sections or figure legends), or explain why this information doesn’t apply to your submission:

Statistical hypothesis and analysis methods are provided into Materials and Methods section.

Detailed Informations about statistics are provided into figure legends and in the “Source data” table file.

When N per group is less than 10, the graph present each sample.

(For large datasets, or papers with a very large number of statistical tests, you may upload a single table file with tests, Ns, etc., with reference to sections in the manuscript.)

**Group allocation**

- Indicate how samples were allocated into experimental groups (in the case of clinical studies, please specify allocation to treatment method); if randomization was used, please also state if restricted randomization was applied
- Indicate if masking was used during group allocation, data collection and/or data analysis

Please outline where this information can be found within the submission (e.g., sections or figure legends), or explain why this information doesn’t apply to your submission:

Not applicable for in vitro studies

For in vivo experiments, animals were allocated into experimental or control groups depending on the genotype.

**Additional data files (“source data”)**

- We encourage you to upload relevant additional data files, such as numerical data that are represented as a graph in a figure, or as a summary table
- Where provided, these should be in the most useful format, and they can be uploaded as “Source data” files linked to a main figure or table
- Include model definition files including the full list of parameters used
- Include code used for data analysis (e.g., R, MatLab)
- Avoid stating that data files are “available upon request”

Please indicate the figures or tables for which source data files have been provided:

All available data are reported in the manuscript.

Figures for which source data files have been provided:

Figure 3

Figure 4

Figure 5

Figure 6

Figure 7

Figure 8

Figure 9

Supplementary Figure 2

Supplementary Figure 3

Supplementary Figure 4

Supplementary Figure 5
